# Mitochondrial ROS-Modulated mtDNA: A Potential Target for Cardiac Aging

**DOI:** 10.1155/2020/9423593

**Published:** 2020-03-26

**Authors:** Yue Quan, Yanguo Xin, Geer Tian, Junteng Zhou, Xiaojing Liu

**Affiliations:** ^1^Laboratory of Cardiovascular Diseases, Regenerative Medicine Research Center, West China Hospital, Sichuan University, Chengdu 610041, China; ^2^Department of Cardiology, West China Hospital, Sichuan University, Chengdu 610041, China; ^3^Laboratory of Mitochondrial Biology, West China-Washington Mitochondria and Metabolism Center, West China Hospital, Sichuan University, Chengdu 610041, China

## Abstract

Mitochondrial DNA (mtDNA) damage is associated with the development of cardiovascular diseases. Cardiac aging plays a central role in cardiovascular diseases. There is accumulating evidence linking cardiac aging to mtDNA damage, including mtDNA mutation and decreased mtDNA copy number. Current wisdom indicates that mtDNA is susceptible to damage by mitochondrial reactive oxygen species (mtROS). This review presents the cellular and molecular mechanisms of cardiac aging, including autophagy, chronic inflammation, mtROS, and mtDNA damage, and the effects of mitochondrial biogenesis and oxidative stress on mtDNA. The importance of nucleoid-associated proteins (Pol *γ*), nuclear respiratory factors (NRF1 and NRF2), the cGAS-STING pathway, and the mitochondrial biogenesis pathway concerning the development of mtDNA damage during cardiac aging is discussed. Thus, the repair of damaged mtDNA provides a potential clinical target for preventing cardiac aging.

## 1. Introduction

Cardiovascular diseases (CVDs) account for 31% of all deaths worldwide [1]. Age is widely recognized as the leading risk factor for CVDs. Cardiac aging is defined as the gradual deterioration of cardiac structure and function with age [2]. Diastolic dysfunction and left ventricular hypertrophy often occur in the elderly. Valvular calcification and fibrosis cause the development of aortic stenosis with age. The ventricular and valvular changes above make the aged heart more vulnerable to stress and contribute to the increased mortality and morbidity of CVDs in the elderly [3, 4]. The aged heart also exhibits a decrease in the number of myocytes, an increase in the size of cardiomyocyte, and an increase in the accumulation of lipids and fibrosis [5]. The interrelationship between the underlying mechanisms of cardiac aging and the interaction between cellular and molecular aging processes and disease-specific pathways are intricate. Elucidating the potential mechanisms of cardiac aging can promote the development of “antiaging” therapies to prevent or delay the cardiovascular changes.

To explore potential targets of heart aging, it is important to obtain knowledge of adequate preclinical models, which can be used to study the mechanisms of cardiac aging. Canine hearts develop myocardial hypertrophy and accumulate lipofuscin and amyloid, leading to increased myocardial stiffness [6]. Because the distribution of the cardiac conduction system and the electrophysiological properties of dogs are similar to those of the human heart, the dog model has been widely used for electrophysiological research [4]. The Drosophila melanogaster heart has a similar molecular structure and basic physiology as the human heart. Both fly and human hearts experience age-related morphological and functional decline. Several genes in mammals that regulate oxidative stress and cardiac hypertrophy also affect the cardiac aging in a fruit fly [7]. Elderly rhesus monkeys exhibit degenerative calcifications of the aortic and mitral valves, myocardial hypertrophy, lipofuscin accumulation, interstitial fibrosis, myocardial infarction, and congestive heart failure [4]. Aged mouse hearts indicate increased fibrosis, amyloid deposition, and increased myocardial fiber size [8]. The systolic and diastolic function are also significantly impaired with age. Aged rat hearts demonstrate cardiomyocyte hypertrophy, increased LV fibrosis, and impairment of systolic and diastolic function [4].

**Figure 1:**
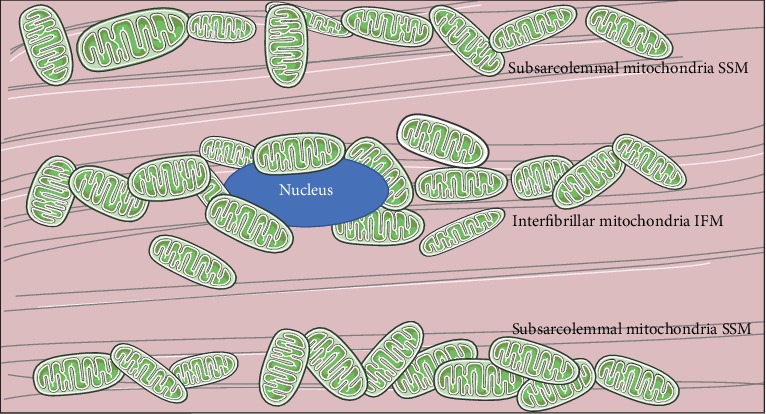
Schematic diagram of cardiomyocyte: the location of the subsarcolemmal mitochondria (SSM) and interfibrillar mitochondria (IFM).

The exact mechanisms involved in cardiac senescence are still not fully understood. Current evidence indicates that cardiac senescence is concerned with dysfunctional organelles with age [9]; meanwhile the decline in mitochondrial function during aging has been reported as a fundamental principle of aging biology for many years. As the “energy house” to fuel normal cardiac function, mitochondria research is not confined to bioenergy; new evidence has revealed unanticipated roles of mitochondria as metabolic transit points and platforms for intracellular signaling that modulate cell activities. Subsarcolemmal mitochondria (SSM) and interfibrillar mitochondria (IFM) (Figure 1) are two structurally similar but biochemically different mitochondrial populations in the heart [10]. Nevertheless, the mitochondrial defects in aging have been limited to the IFM population.

Mitochondrial-derived oxidative stress plays a vital role in cardiac aging through irreversible damage to mitochondrial DNA (mtDNA). Enzymes of the electron transport system reside on the inner mitochondrial membrane (IMM), encompassing the mitochondrial matrix containing mtDNA. Excessive mitochondrial reactive oxygen species (mtROS) can damage DNA [11, 12]. The mtDNA damage, which reduces the stabilization of adequate ATP supply during cardiac aging, disrupts the balance of cellular apoptosis, mitochondrial bioenergetics, and biogenesis.

Mitochondrial biogenesis (MB) is the underlying mechanism that controls the number of mitochondria. Mitochondrial function is strongly dependent on the morphology of the mitochondria [13]. Changes in the shape, number, and localization of the mitochondria can cause significant functional modifications. Mitochondria are dynamic entities that undergo movement, fission, and fusion processes, collectively termed the “mitochondrial dynamism”; the morphology plays critical roles in apoptosis, cell death, and development [14, 15]. The coordinated expression of the mitochondrial genome and the nuclear genes encoding mitochondrial proteins is involved in mitochondrial biogenesis. In this review, we highlight the specific mtDNA linked to mitochondrial biogenesis, oxidative metabolism, and the latent clinical utility of mtDNA in the aged heart.

## 2. Molecular and Cellular Mechanisms of Cardiac Aging

Aging is a complex process via many molecular and cellular mechanisms contributing to a dysfunction in organ function. Better understanding of the mechanisms involved in cardiac aging can guide us to promote healthy heart aging and mitigate the burden of CVD in the elderly. The major mechanisms involved in alterations in the heart are mitochondrial dysfunction, altered autophagy, chronic inflammation, increased mitochondrial oxidative stress, and increased mtDNA instability.

### 2.1. Inflammation in Cardiac Aging

Inflammation is a hallmark in the cardiac aging process. Inflammatory processes, especially those that mediate chronic low-grade inflammation, are known to lead to the development of age-related CVDs [16]. Mitochondrial dysfunction is closely associated with immune response and chronic inflammation. Studies support that mtROS contribute to the inflammation in the cardiovascular system [17]. mtROS activate the redox-sensitive mediator, nuclear factor-*κ*B (NF-*κ*B), which regulates the transcription of various proinflammatory cytokines [11, 18]. As the heart ages, prolonged exposure to high levels of oxidants leads to the activation of NF-*κ*B-mediated inflammation. Mitochondrial dysfunction leads to the leak of mtDNA in the cytoplasm or even in the circulation, which can be sensed by toll-like receptor 9 (TLR9) [19]. TLR9 is critical for the synthesis of proinflammatory cytokines. Thus, the leaking mtDNA activates caspase-1 and promotes the secretion of IL-1*β* and IL-18 in macrophages. In the aged heart, the increased senescent cells modulate inflammation through secreting chemokine and cytokines, such as IL-1*β*, IL-6, and IL-8, termed the senescence-associated secretory phenotype (SASP) [19, 20]. The cyclic GMP-AMP synthase (cGAS) is a stimulator of the interferon genes (STING). As a DNA sensor, cGAS combines with cytosolic DNA, inducing the production of cyclic GMP-AMP (cGAMP), which activates STING. Activated STING causes the interferon-regulatory factor 3 (IRF3) transcription factor to enter the nucleus, resulting in the secretion of interferon (IFN). The cGAS-STING signaling pathway has been identified as a SASP regulator [21, 22]. When mtDNA is released into the cytoplasm, inflammation is activated through the SASP program initiated by cGAS-STING [23]. Together, the activation of age-related inflammatory processes plays a key role in cardiac aging while cGAS-STING signaling regulates inflammation via multiple mechanisms, which might be a novel intervention target.

### 2.2. Autophagy in Cardiac Aging

Autophagy is an important cellular process involved in aging and longevity that gradually declines during cardiac aging, resulting in an increase in the sensitivity of the heart to stress [24]. Autophagy is a catabolic process involved in lifespan and aging in the cardiovascular system [25]. It plays a pivotal role in the degradation of damaged or long-lived organelles and proteins though lysosomes. Autophagy and autophagic flux are blocked in the aged heart, resulting in greater susceptibility to stress. The three known types of autophagy are chaperone-mediated autophagy (CMA), microautophagy, and macroautophagy [26]. Macroautophagy, referred to as autophagy, is the most studied autophagic process. Macroautophagy begins with a small vesicular sac, called the phagophore. The phagophore encloses long-lived cytosolic proteins and organelles, forming a double-membraned structure termed an autophagosome [27]. Then, the autophagosomes fuse with lysosomes to form autophagolysosomes, where the cargo is degraded to provide substrates for cellular metabolism to maintain cellular homeostasis. Microautophagy involves the direct capture and engulfment of cytoplasmic cargo through invaginations of the lysosomal membrane. Parts of the damaged mitochondria are degraded by microautophagy [3]. CMA is a highly selective process that specifically targets the cytosolic proteins with a KFERQ motif for degradation.

Autophagy-related genes (ATG) are required for the formation of autophagosomes in yeast. ULK1, the mammalian homologue of ATG1, performs a similar function. Serine/threonine kinase, the mammalian target of rapamycin (mTOR) is the main regulator of autophagy negatively regulated by mTOR complex 1 (mTORC1) [28]. mTORC1 regulates autophagy induced by rapamycin and changes in nutritional and energy status through ULK1-Atg13-FIP200 complex in mammals [29]. Upon nutrient starvation, mTORC1 is inactivated, thus relieving the inhibition of phosphorylation of ULK1-Atg13-FIP200 complex. In cardiac aging, the modulation of autophagy involves AMP-activated protein kinase (AMPK) [2]. Activated AMPK turns on autophagy though the inhibition of mTOR and redirects metabolism towards increased catabolism and decreased anabolism. During aging, AMPK-mediated autophagy is reduced, suggesting cardiac dysfunction. In addition, AMPK phosphorylation of Ulk1 at a specific serine residue leads to the initiation of autophagy [30]. Rapamycin, an mTOR inhibitor and autophagy inducer, reverses cardiac remodeling and contractile dysfunction without affecting the inflammation state of the elderly heart. Rapamycin feeding for 10 weeks induces autophagy, ameliorates energy metabolism, and alters the myocardial metabolome in aged female mice [5]. As a downstream regulator of AMPK, mTOR plays a crucial role in senescence-induced cardiac remodeling. Resveratrol, an AMPK activator, inhibits mTOR with an antiaging effect. Rapamycin and resveratrol, both of which can activate autophagy, are beneficial for the treatment of cardiac remodeling and heart dysfunction [31]. This emerging evidence suggests that autophagy plays a nonnegligible cardioprotective role with clinical connotations.

## 3. Mitochondrial Dysfunction in Cardiac Aging

The heart has high energy demand and a high density of mitochondria. Decreased energetic capacity of the cardiac mitochondria is related to aging [32], and the heart is particularly vulnerable to mitochondrial dysfunction caused by damaged structures and increased ROS. Mechanisms contributing to disrupted bioenergetics include decreased nicotinamide adenine dinucleotide (NAD^+^) levels [33], reduced efficacy of the respiratory chain, mutated mtDNA, leaking electrons, and dysregulated mitochondrial biogenesis. The regular action of membrane transport and barrier functions depends on cellular energy metabolism; thus, the damaged mitochondrial energy metabolism causes decreased electron transport chain (ETC) function and increased ROS generation.

Evidence suggests that the mitochondria structure is disrupted in cardiac senescence, with increased mitochondria size [34]. Electron-microscopy-based studies have demonstrated that the area of IMM obviously decreases [35], showing a loss of cristae with age in rodent heart [36]. Mitochondrial dynamics have been involved in the aging process [37], and the promotion of fusion or blockade of fission prompts cell senescence [17]. Mitofusins (MFN)-1/2 and optic atrophy protein-1 (OPA1) regulate mitochondrial morphology inside adult cardiomyocytes [38]. The deleterious effects of stress-induced OPA1 processing on myocardial function reveal the link between cardiac metabolism and mitochondrial dynamics [39]. The mitochondrial area and ultrastructure are deranged in heart failure with reduced ejection fraction (HFrEF), where the markers of mitochondrial fission dynamin-related protein-1 (DRP1) are deranged [40]. The balance between fusion and fission is crucial to maintaining heart health [41].

An imbalance between fission and fusion is detrimental to mitochondrial homeostasis and mitochondrial quality [2]. Hence, addressing this unfavorable situation is a vital issue. Mitochondrial fragmentation and damaged mitochondria can be cleared by a form of selective autophagy-mitophagy. Mitophagy is a specific class of autophagy eliminating dysfunctional mitochondria from the heart under normal physiological conditions and pathological stresses, maintaining healthy mitochondria at a stable number [42]. Mitochondrial fusion and mitophagy were observably suppressed by ischemia-reperfusion (I/R) injury, accompanied by myocardial inflammation, infarction area expansion, heart dysfunction, and cardiomyocyte oxidative stress [43]. The mitochondrial membrane kinase, PTEN-induced kinase-1 (PINK1), and the cytosolic E3 ubiquitin ligase (Parkin) pathway are the major mitophagy pathways in mammalian cells [23, 44]. The damaged mitochondria are sensed by the decreased mitochondrial membrane potential (*ΔΨ*m) and transduced to Parkin via the autophosphorylation of PINK1 [45]. Ubiquitination of mitochondrial outer membrane proteins mediated by Parkin is an initial signal for autophagosome phagocytosis and subsequently progresses to lysosome degradation [42]. There were severe defects in mitochondrial homeostasis in PINK1 KO mice accompanied by changes in the mitochondrial network and an increase in ROS [46]. Parkin and PINK1 prevent inflammation by removing damaged mitochondria, thereby preventing the increase in cytosolic and circulating mtDNA and providing a new model for how mitophagy may mitigate CVDs [23]. The increase in mitochondrial damage and the decrease in mitochondrial metabolism due to impaired and deficient of mitophagy can lead to the accumulation of damaged mitochondria in cells and aggravate the process of cardiac aging.

## 4. Mitochondrial Oxidative Metabolism and mtDNA Mutation in Cardiac Aging

### 4.1. Production of Oxidants in the Aging Heart

Nohl and Hegner [47] discovered that heart mitochondria in old rats generated more H_2_O_2_ than did mitochondria from the young *in vivo*. Since then, a large body of studies have been published to support the role of mitochondria and cardiac mitochondrial oxidant production in the aging process, identifying that the production of intramitochondrial ROS is the major determinant of aging [48, 49]. In aging, mitochondria produce the majority of ROS during oxidative phosphorylation (OXPHOS) and ATP generation [50]. Deficient electron transport chains (ETCs) are a potential site for ROS production, including subunit complexes I and III [51, 52]. The mitochondrial free radical aging theory hypothesizes that age-related increases in mitochondrial ROS lead to mtDNA mutations and the accumulation of oxidative protein and lipid, which reduce mitochondrial respiratory efficiency [53]. Under homeostatic physiological conditions, a large amount of superoxide anion (•O^2-^) is generated through oxygen transformation due to the leaking of electrons mainly from complexes I and III [54, 55], and mitochondrial manganese superoxide dismutase (SOD2) converts •O^2-^ into H_2_O_2_ [56]. The increased release of H_2_O_2_ activates NF-*κ*B-mediated inflammatory response and mitochondrial dysfunction during aging. H_2_O_2_ is then catabolized by glutathione peroxidase I (GPX1) and catalase (CAT). GPX1 reduces H_2_O_2_ to glutathione and water. CAT is a common enzyme that catalyzes H_2_O_2_ to water and oxygen. CAT largely determines mitochondrial antioxidant capacity and is the enzyme most affected during aging [57].

**Figure 2:**
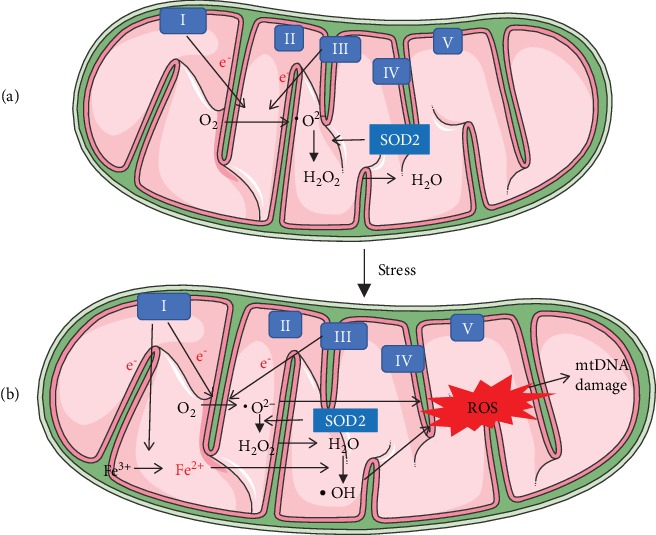
Production of oxidants. (a) Under normal physiological conditions, the leaking of electrons from complexes I and III generates highly toxic •O^2-^ through oxygen transformation, and •O^2-^ is converted into less toxic H_2_O_2_ by SOD2 and neutralized into O_2_ and H_2_O. In the presence of iron, H_2_O_2_ is converted into •OH. (b) Under pathological conditions, the increase in mitochondrial iron accelerates the generation of •OH and ROS in mitochondria. mtROS can lead to mtDNA damage.

Iron is stored in ferric (Fe^3+^) form inside ferritin. Oxidative damage to ferritin can cause the release of redox-active ferrous (Fe^2+^) iron. mtROS-derived mtDNA damage results in a decrease in mitochondrial membrane potential. The reduced mitochondrial membrane potential contributes to the defective transport of iron-sulfur proteins into and out of mitochondria, which is important for the assembly of the mitochondrial iron-sulfur cluster (ISC) and the maturation of iron-sulfur proteins. Defects in the mitochondrial ISC machinery lead to impaired iron homeostasis with increased iron accumulation in mitochondria [58]. In the presence of Fe^2+^, H_2_O_2_ is converted into the highly reactive hydroxyl radical (•OH) [59]. The mitochondrial iron content increases with aging in the myocardium, which accelerates the generation of •OH and oxidative damage in aging [60]. In old rats, the rate of generation of •O^2--^ and •OH anion radicals is significantly increased in heart mitochondria [61] (Figure 2).

ROS plays a pivotal role in healthy cellular and mitochondrial signaling and functionality. However, if unchecked, ROS can mediate oxidative damage to tissues and cells, leading to a vicious cycle of inflammation and more oxidative stress. Meanwhile, mitochondria, the major source of ROS, are thought to be particularly vulnerable to oxidative damage. Because of its richness in mitochondria and high oxygen demand, the heart is at high risk of oxidative damage. The most supportive evidence of the central role of mtROS in the aged heart is that overexpression of catalase targeted to mitochondria (mCAT) attenuates cardiac aging [62]. mCAT mice are resistant to fibrosis, cardiac hypertrophy, and biogenesis as well as heart failure [63]. ROS destroys myocardial energetics, leading to the decreased contractile reserve and slowed relaxation. mCAT can correct these effects preceding structural remodeling, suggesting that ROS-mediated energetic damage is sufficient to cause contractile dysfunction in the metabolic heart [64].

### 4.2. Mitochondrial Oxidative Stress and mtDNA Mutation in Cardiac Aging

A growing body of evidence suggests that there is increasing oxidative damage to mitochondrial DNA in cardiac aging [65, 66]. Because of the histone deficiency, limited DNA repair capabilities, and proximity of mtDNA to the site of mtROS generation, mtDNA can suffer various types of damage, including mtDNA point mutations, mtDNA point deletions, and decreased mtDNA copy number (mtDNA-CN) [67]. The oxidative damage to mtDNA has different types, including single-strand breaks (SSBs), double-strand breaks (DSBs), and oxidized bases such as 7,8-dihydro-8-oxoguanine (8oxoG). The continuous replicative state of mtDNA and existence of the nucleoid structure render mitochondria vulnerable to oxidative damage and mutations. Single-stranded DNA-binding protein (SSB), transcription factor A (TFAM), RNA polymerase (POLRMT), DNA polymerase gamma (Pol *γ*), and Twinkle helicase are the primary nucleoid-associated proteins in mitochondria [68, 69]. TFAM and DNA Pol *γ* are the two crucial metabolism-related genes. Their deletion or overexpression can promote the development of heart failure (HF) in transgenic mice [67]. Homozygous mutation of mitochondrial polymerase *γ* (Pol*g*^*m*/*m*^) in mice causes cardiac hypertrophy, accelerates aging, and accumulates mutations and deletions of mtDNA [70]. 8oxoG is a common mtDNA oxidation product, and it is considered to be a cellular marker of DNA damage induced by oxidative stress [65]. Previous *in vitro* studies suggest that TFAM preferentially binds to 8oxoG to hinder the repair processes [71]. The removal of 8oxoG is a multistep process that depends on the proteins encoded by mutY DNA glycosylase (*MUTYH*) and 8oxoG DNA glycosylase (*OGG1*) genes. MUTYH excises the misincorporating adenine-inserted opposite 8oxoG [72]. In the human mitochondria, OGG1 excises 8oxoG mispaired with adenine efficiently by catalyzing the splitting of an N-glycosidic bond between the damaged 8oxoG base and a deoxyribose sugar. OGG1 is the main enzyme for base excision repair (BER) of 8oxoG lesions [73]. DNA Pol *γ* plays a vital role in mtDNA replication [62] simultaneously involving Twinkle helicase and SSB. DNA Pol *γ* has two main functions: mtDNA synthesis and proofreading. Recent studies report that ROS reduces the proofreading ability of Pol *γ*, causing replication errors. Thus, oxidation aggravating mtDNA mutations causes replication errors, which indirectly cause mtDNA damage [65, 74]. This proves that mtDNA mutations are largely random rather than transversional, and Pol *γ* oxidation is likely to account for mtDNA mutations in aging. Therefore, mtDNA mutation may be highly associated with heart aging.

### 4.3. Oxidative Damage to Mitochondrial DNA Copy Number

Altered mtDNA copy number (mtDNA-CN) and increased mutations render impaired mtDNA integrity, causing cellular dysfunction during aging [75]. A calculation of mtDNA-CN by the relative ratio of DNA from the mitochondrial gene NADH dehydrogenase subunit to the nuclear gene cytochrome P4501A1 found that mtDNA-CN decreased in angiotensin (Ang) II-induced cardiac hypertrophy mice [70]. mtDNA-CN is inversely associated with both prevalence and incidence in CVDs and sudden cardiac death (SCD) [76, 77]. mtDNA-CN can be an indirect biomarker of mitochondrial function. Its decline in cells indicates a concomitantly reduced energy metabolism, which may indicate the lack of oxidative stress response. The oxidative stress response causes damage to mtDNA replication enzymes and thus aggravates the decrease in mtDNA-CN further [78]. In pressure-overload-induced HF mice, increased mtDNA-CN induced by the overexpression of Twinkle or TFAM-alleviated fibrosis of the left ventricle, limited mitochondrial oxidative stress, and improved cardiac function [79, 80]. One study observed an inverse association between mtDNA-CN and coronary artery disease in a Chinese population, especially among smokers, and found an inverse correlation between mtDNA-CN and ROS production. This study indicates a vital relationship among mtDNA-CN, oxidative stress, and coronary artery disease [81]. This evidence suggests that mtDNA-CN has potential clinical utility in improving heart damage.

## 5. MtDNA Homeostasis Associated with the Mitochondrial Biogenesis in Cardiac Aging

### 5.1. Mitochondrial Biogenesis Pathway

Mitochondrial biogenesis (MB) is the basis of the mitochondrial life cycle, including coordinated synthesis of nuclear DNA- (nDNA-) and mtDNA-encoded proteins, mtDNA replication, transcription of mitochondrial RNA (mtRNA), and translation of mitochondrial mRNAs. To ensure the proper assembly and function of a large number of proteins assembling the mitochondrial respiratory chain, MB requires the coordination of nDNA- and mtDNA-encoded gene expression [82]. The increased MB in cardiac aging is considered to be a compensatory maladaptive response to the damaged energy metabolism, which is also stimulated by age-related mtROS. Decreased MB is a vital mechanism responsible for myocardial injury and HF [83, 84]. MB alleviates mitochondrial dysfunction induced by oxidative stress and thus is considered to be a novel repair mechanism in aged heart.

**Figure 3:**
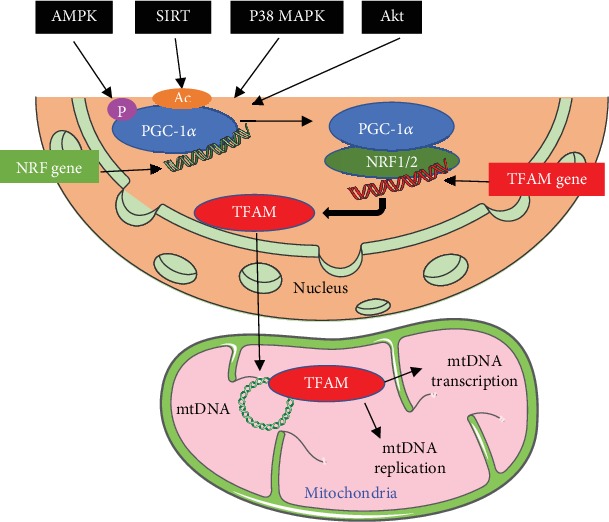
The regulation of mitochondrial biogenesis by the PGC-1*α*-NRF1-TFAM pathway. PGC-1*α* is activated via phosphorylation by AMPK, deacetylation by Sirtuin, and p38 MAPK. Activated PGC-1*α* and NRF1/2 result in the synthesis of TFAM. TFAM is a mitochondrial transcriptional regulator encoded by nDNA. Then, TFAM is imported into mitochondria to stabilize mtDNA and enhance the synthesis of subunits of ETC encoded by mtDNA, leading to transcription and replication of mtDNA.

Nuclear respiratory factors (NRF1/2) and the peroxisome proliferator-activated receptor gamma coactivator-1*α* (PGC-1*α*) regulate the expression of nDNA encoding mitochondrial proteins that are required for respiratory complex and biological function, including fatty acid oxidation (FAO), OXPHOS, and electron transport chain (ETC) [85, 86]. PGC-1*α* modulates the expression of nDNA-encoded genes, such as TFAM, by interacting with NRF1/2 in mtDNA promoters [33]. Meanwhile, TFAM works in conjunction with mtRNA polymerase to confer promoters with specificity and to increase the transcription initiation rate of mtDNA genes. This process executes replication, transcription, and translation of mtDNA [87, 88] (Figure 3). Research suggests that enalapril reduced mtROS-derived damage and cardiac hypertrophy. Following enalapril treatment, the binding of TFAM to mtDNA regions involved in transcription and replication became stronger in old rats. Mitochondrial mass, autophagy, and MB also increased in enalapril-treated rats [89]. The increased protein levels of NRF1 and TFAM, which are mitochondrial biogenesis factors, caused the restoration of mtDNA loss by oxidants [83]. Taken together, the increased MB may be a therapeutic strategy for heart injury.

### 5.2. Regulation of Mitochondrial Biogenesis

Many studies indicate that PGC-1*α* activation through genetic or drug intervention can prevent telomere shortening and age-related changes in the heart [90, 91]. Decreased PGC-1*α* is a common characteristic in various cardiovascular diseases in mice [92, 93]. PGC-1*α* has emerged as a powerful regulator of mitochondrial biology in the heart and serves as a master regulator of MB and mitochondrial function [94]. At the posttranslational level, the PGC-1*α* activity is regulated via phosphorylation by some signaling pathways, including Akt (protein kinase B), AMPK, deacetylation of Sirtuin (SIRT1/3) [95], and mitogen-activated protein kinase (MAPK) p38 (Figure 3).

Intracellular Ca^2+^ handling was impaired with advanced aging. Excessive accumulated Ca^2+^ in mitochondria not only leads to damage of the oxidation respiratory chain, decreased MB, and increased mtROS but also causes mitochondrial dysfunction, cell apoptosis, and death [96]. The p38 MAPK pathway was activated and induced calcium overload during I/R, which could be relieved by SB203580 (an inhibitor of p38 MAPK) to accelerate the recovery speed of mitochondrial biogenesis and to increase the mtDNA content [97]. Reducing mitochondrial ROS by mitochondria-targeted antioxidant peptide attenuated Ang-induced mitochondrial oxidative damage, decreased MB, increased the phosphorylation of p38 MAPK, and then improved Ang-induced cardiac hypertrophy and fibrosis [98].

**Figure 4:**
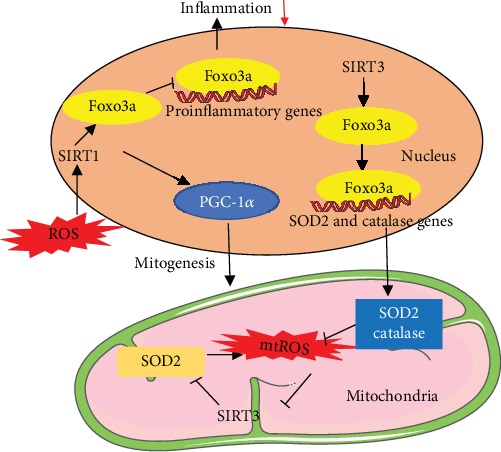
Crucial roles of SIRT1 and SIRT3 in regulation of mitochondrial biogenesis and oxidative stress. SIRT1 is located in the nuclei and regulates mitochondrial functions by deacetylating Foxo3a and attenuating its function to reduce the expression of inflammatory proteins. SIRT1 activates PGC-1*α* by deacetylating the lysine residues to induce mitochondrial biogenesis. Oxidative stress inactivates SIRT3, resulting in the inactivation of SOD2 hyperacetylation and induction of mtROS. This forms a vicious cycle between mitochondrial dysfunction and mitochondrial oxidative stress. The increased ROS can be reduced by SIRT3-mediated deacetylation and activation of Foxo3a and SOD2. Deacetylated Foxo3a enhances the expression of antioxidant genes SOD2 and catalase to reduce mtROS.

SIRT1 and SIRT3, located in the nuclei and mitochondria, regulate mitochondrial functions by deacetylation of nuclear proteins and mitochondrial proteins, respectively. SIRT1 is expressed abundantly in mammalian hearts. It is an NAD^+^-dependent deacetylase and a marker of MB [99]. Activated SIRT1 improves mitochondrial dysfunction and ameliorates cardiac defects in diabetic animals. SIRT1 promotes MB though deacetylation and activation PGC-1*α*, thereby completing the metabolic pathway and inhibiting inflammatory signaling [100]. SIRT1-deficient primary myoblasts reduce the mtDNA content and mitochondrial membrane potential. SIRT1 deletion increases both mtROS and the rate of oxidative damage. After pressure overload, SIRT1 gene deletion mice have exhibited exacerbated cardiac dysfunction and alterations of mitochondrial properties [101]. Melatonin ameliorates myocardial I/R injury via SIRT1 activation [99]. SIRT3 has been considered a crucial mitochondrial deacetylase, playing a vital role in energy production, including the supply of intermediates for tricarboxylic acid cycle (TCA) and ETC activation [102]. Oxidative stress inactivates SIRT3 by S-glutathionylation, resulting in inactivation of SOD2 hyperacetylation and induction of mtROS. This forms a vicious cycle between mitochondrial dysfunction and mitochondrial oxidative stress [56]. The increased ROS can be reduced by SIRT3-mediated deacetylation and activation of transcription factor forkhead box O3a (Foxo3a). Deacetylated Foxo3a enhances antioxidant genes SOD2 and catalase, thereby reducing mtROS to protect cardiac function [103]. Under oxidative stress conditions, the Foxo3a existing in the nucleus induces the expression of inflammatory proteins. SIRT1 protects the cell and stabilizes nDNA by deacetylating Foxo3a and attenuating its function [104] (Figure 4). SRT1720, an activator of SIRT1, ameliorates contractile dysfunction and impaired mitophagy in cardiac aging [105]. SIRT3-deficient mice are more susceptible to age-dependent cardiac hypertrophy [106]. Doxorubicin (Doxo), a widely used clinical cancer drug, has a severe side effect on the heart. One study demonstrated that SIRT3 activation protected the heart from Doxo-induced cardiotoxicity by repairing mtDNA damage [66]. Upregulation of SIRT1/3 may improve age-induced cardiac dysfunction, suggesting the therapeutic potential of SIRT1/3 in cardiac aging.

AMPK is an essential cellular fuel sensor of cellular energy defects and controls mitochondrial biogenesis, myocardial morphology, and contractile function. AMPK deficiency may be associated with age-induced cardiac dysfunction according to the evidence that AMPK deficiency distinctly enhances age-associated ROS generation [107]. Mitochondrial insult or defect activates AMPK, including mtDNA depletion or mutation, and impairs mitochondrial function and mitochondrial production of ATP [108]. The mitochondrial permeability transition pore (mPTP) opening is a sentinel event that triggers cell death in the early ischemic-reperfusion period. AMPK regulates MB by phosphorylating PGC-1*α*. The overexpression of the active AMPK *γ*3 subunit increased the expression of PGC-1*α* [109]. PGC-1*α* is regulated by AMPK via a variety of indirect mechanisms including p38 MAPK and SIRT1 [108]. Metformin, an AMPK activator at low dose, alleviates age-induced cardiomyocyte contractile defects via inhibition of complex I activity and activation of autophagy and leads to improved MB by increasing PGC-1*α* expression during I/R and heart failure [110, 111]. These studies indicate that the use of metformin should not be limited to the treatment of diabetes mellitus, and it may have potential clinical use for cardiovascular diseases.

## 6. Summary and Conclusions

Cardiac aging resulting in defects in cardiac mitochondrial function centers on the mtDNA damage. The mechanisms of the alterations in the aging heart mainly involve mitochondrial dysfunction, altered autophagy, chronic inflammation, increased mitochondrial oxidative stress, and increased mtDNA instability.

Age-altered mtROS triggers accumulation of point mutations, deletion in mtDNA, and a decrease in mtDNA-CN, leading to impaired mitochondrial function and cell death. TFAM and DNA Pol *γ* are the two critical nucleoid-associated proteins involved mtDNA replication and repair. Oxidative Pol *γ* is likely to interpret mtDNA mutations in cardiac aging. Mitochondrial biogenesis is the basis of the mitochondrial life cycle, including coordinated synthesis of nDNA- and mtDNA-encoded proteins. The increased MB in cardiac aging is a compensatory maladaptive response to the mtROS-induced damaged energy metabolism. NRF1, NRF2, and PGC-1*α* regulate mitochondrial proteins that are essential for respiratory complex expression and biological function. PGC-1*α* activity is regulated via phosphorylation by some signaling pathways, including AMPK, deacetylation of Sirtuin, and MAPK p38.

A low dose of metformin, as an AMPK activator, can prolong the life span of mice without metabolic disorders. Rapamycin prevents cardiac senescence though the inhibition of mTOR. Resveratrol can induce autophagy and increase longevity. Melatonin ameliorates myocardial I/R injury via SIRT1 activation. It is worth noting that these emerging data have important theoretical and practical significance. These conventional clinical drugs can be used to prevent cardiac aging by preventing mitochondrial dysfunction and mtDNA damage. This review provides new insights into mtDNA in cardiac aging. Further research on the mechanisms of mtDNA decline in heart aging is warranted to create an opportunity to develop novel therapies to treat cardiovascular diseases and slow the rate of age-induced heart changes, thus contributing to better outcomes for longevity.
